# Tuberculosis incidence inequalities and its social determinants in Manaus from 2007 to 2016

**DOI:** 10.1186/s12939-018-0900-3

**Published:** 2018-12-29

**Authors:** Daniel Barros de Castro, Elvira Maria Godinho de Seixas Maciel, Megumi Sadahiro, Rosemary Costa Pinto, Bernardino Cláudio de Albuquerque, José Ueleres Braga

**Affiliations:** 1 Fundação de Vigilância em Saúde do Amazonas, Manaus, Brazil; 2 Escola Nacional de Saúde Pública Sérgio Arouca – Fiocruz, Rio de Janeiro, Brazil; 3 Instituto de Medicina Social – UERJ, Rio de Janeiro, Brazil; 4 PECTI-SAÚDE / Fundação de Amparo a Pesquisa do estado do Amazonas, Manaus, Brazil

**Keywords:** Healthcare disparities, Socioeconomic level, Tuberculosis, Brazil

## Abstract

**Background:**

Brazil is among the 30 countries with high-burden of tuberculosis worldwide, and Manaus is the capital with the highest tuberculosis incidence. The accelerated economic and population growth in Manaus in the last 30 years has strengthened the process of social stratification that may result in population groups that are less favored in terms of healthcare and are vulnerable to infection and illness due to tuberculosis. This study aimed to characterize inequalities associated with tuberculosis incidence in relation to the socioeconomic and demographic characteristics of the resident population of Manaus and to identify their determinants from 2007 to 2016.

**Methods:**

An ecological study was conducted using the data from the Diseases Notification Information System. Tuberculosis incidence rates by population characteristics (gender, ethnicity, and socioeconomic level) were calculated for each year, studied, and represented in *equiplot* charts. To measure the disparity of tuberculosis incidence in the resident population in Manaus, the Gini index of tuberculosis in each neighborhood was calculated based on the incidence rates of the census sectors. A thematic map was constructed to represent the spatial distribution of tuberculosis incidence inequality. Linear regression models were used to identify the relationship between the tuberculosis incidence inequality and its social determinants.

**Results:**

From 2007 to 2016, there was an increase in the tuberculosis incidence in Manaus, together with an increase in incident inequality among genders, ethnic groups, and socioeconomic level. The incidence of tuberculosis inequality was associated with the inequalities of its possible determinants (Gini of the proportion of male population, Gini of the proportion of indigenous population, Gini of the proportion of illiteracy, Gini of income, Gini of the proportion of households connected to the water network, and Gini of the mean number of bathrooms per inhabitant), the per capita income, and the proportion of cases with laboratory confirmation.

**Conclusions:**

Disparities in tuberculosis incidence in the resident population in neighborhoods can be explained by the sociodemographic and economic heterogeneity. Our findings recommend that public policies and tuberculosis control strategies consider differences in the determinants of tuberculosis inequality for the development of specific actions for each population group.

## Background

Tuberculosis (TB) is the ninth leading cause of death worldwide and the leading cause from a single infectious agent. The TB incidence varies substantially across countries and across different population groups within countries. In 2016, of the 1.47 million deaths due to TB in the world, half occurred in three countries: India, Nigeria, and Indonesia. In that year, it is estimated that 10.4 million new cases have emerged, 56% of them in five countries: India, China, Indonesia, the Philippines, and Pakistan [1].

Brazil is among the 30 countries with high-burden of tuberculosis worldwide [1]. Amazonas is a Brazilian state with the highest incidence rate, with 74.1 cases per 100,000 inhabitants, in 2017 [2]. Manaus, the capital state, accounts for 70% of the TB cases in Amazonas [3]. In 2017, the TB incidence rate in Manaus was 104.7 cases per 100,000 inhabitants, the highest among Brazilian capitals [2].

A large number of studies have highlighted the relationship between TB and socioeconomic level in different populations [4, 5]. Recently, researchers have suggested that, as important as absolute poverty, the disease concentration and its transmission in vulnerable populations may explain why reduction in TB epidemics has been slow. This scenario is observed despite the success of the directly observed treatment strategy (DOTS) program’s global implementation and optimistic forecasts of epidemiological models that ignore the heterogeneity of disease occurrence in the population [6, 7].

Health inequalities refer to the differences in incidence, prevalence, mortality, disease burden, and other adverse health conditions that exist among specific population groups [8, 9]. Woodward A and Kawachi I [10] argued that it is important to reduce health inequalities because they are unfair and preventable. In addition, reducing inequalities benefits the whole population and is the most efficient way to control some diseases. Using a mathematical model, Andrews JR, Basu S, Dowdy DW and Murray MB [7] compared the effects of an intervention program, aimed at doubling the TB diagnosis rates, implemented homogeneously throughout the population and those directed toward the richest or poorest group of the population. This study showed that the impact of the intervention on the tuberculosis prevalence in the poorest population is 27% higher than the non-targeted intervention, while the impact of the intervention on the richest population was 23% lower [7].

Scenarios where there is greater disparity in TB incidence may require more resources to achieve the same impact of disease control measures in the population than those with less heterogeneity. Thus, it is important to know the magnitude of these disparities and to recognize population groups with high disease burden. However, knowledge on the TB social determinants does not seem sufficient to recognize the magnitude of disease incidence inequalities in Manaus nor its relationship with social inequities.

It is a challenge for public health researchers working with patients who have TB to generate knowledge about the discrepancies in TB incidence. It should be emphasized that this knowledge is fundamental to subsidize the formulation of public policies with a vision of planning effective TB control actions, reducing inequities and improving the population’s health conditions. Hence, the present study aimed to characterize the TB incidence inequality in the demographic and socioeconomic level and to evaluate its relation to the social determinants in Manaus.

## Methods

A mixed ecological study was carried out, with the neighborhoods of the municipality of Manaus as the units of spatial analysis and the calendar year as temporal unit.

**Fig. 1:**
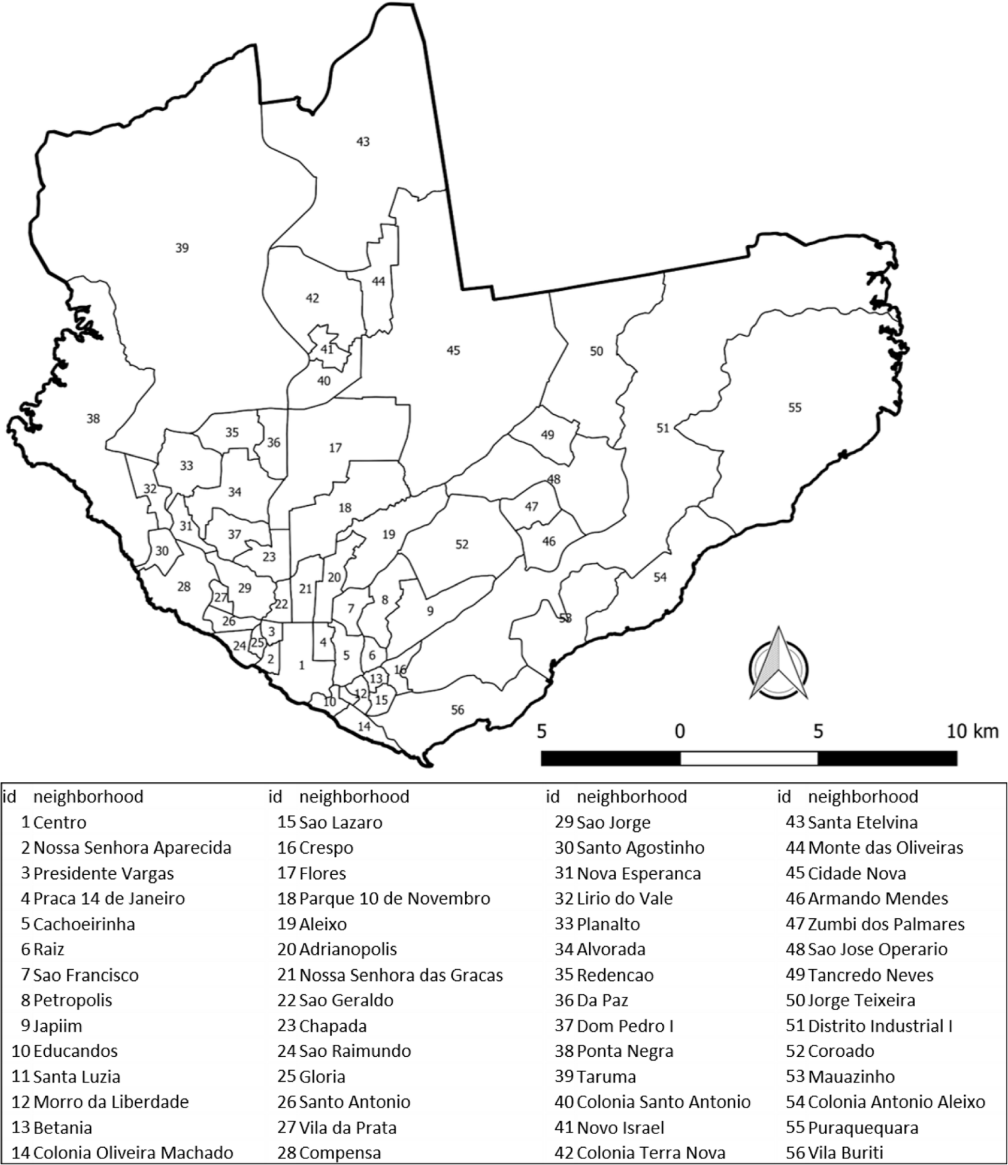
Neighborhoods of Manaus

Manaus (south, 3°6′26″; west, 60°1′34″), the capital of the state of Amazonas, is an important economic and corporate center of the north. In 2008, the urban area was composed of 56 neighborhoods, as shown in Fig. 1. The seven neighborhoods created in 2010 were kept in their original neighborhoods, as follows: Cidade de Deus, Nova Cidade, Novo Aleixo, and Gilberto Mestrinho were considered as Cidade Nova neighborhood; Tarumã Açu as Tarumã; Distrito Industrial II as Distrito Industrial; and Lago Azul as Santa Etelvina. The population of this municipality represented 53% of the inhabitants of the state.

Individual data from TB cases were obtained from the Disease Notification Information System provided by the Amazonas Health Surveillance Foundation. Data regarding gender, ethnicity, and patient’s place of residence were extracted from each registry. All new cases of pulmonary TB affecting the residents in Manaus, regardless of gender or age, were reported from January 1, 2007 to December 31, 2016. The new case definition advocated by the Brazilian Ministry of Health [11] was used. Cases with duplicate records were excluded from this study as well as those with closure status due to a “change in diagnosis,” as these are not cases of TB.

To characterize the socioeconomic situation of the neighborhoods’ population, the municipal human development index for the year 2010, made available by the United Nations Development Program [12], was used. The resident population in the neighborhoods was obtained from the censuses carried out by the Brazilian Institute of Geography and Statistics (called IBGE) in 2000 and 2010. From these data, the populations for the inter-census years were estimated using a linear interpolation technique. The cartographic digital meshes containing the delimitation of the census sectors and neighborhoods of Manaus were obtained from IBGE.

To analyze the TB incidence inequality in the subgroups (gender and ethnic strata), the TB incidence rates in men and women as well as in non-indigenous (white, brown, yellow, and black) and indigenous groups were calculated for each year studied. To analyze the incidence of TB inequality in relation to the socioeconomic level, the neighborhoods of Manaus were classified according to quintiles of HDI. For the worst and best groups of neighborhoods in level of human development (1st and 5th inter-quintile intervals, respectively), the annual TB incidence rates were calculated. The difference between the incidence rates of these groups was showed by means of *equiplot* charts. *Equiplot* is a graphical representation widely used in health equity studies and is recommended by the *International Center for Equity in Health* at the Federal University of Pelotas (www.equidade.org/equiplot) [13, 14].

To analyze the TB incidence inequality in Manaus, the Gini index of the TB incidence for each neighborhood was calculated, as was done by other investigations [15–17]. For this, the following were performed: (i) georeferencing of TB cases; (ii) calculation of the mean annual TB incidence rate by census sectors; (iii) calculation of the Gini index of the TB incidence for each neighborhood, based on the incidence rates of the census sectors; and (iv) representation of the spatial distribution of TB Gini in neighborhoods by thematic map.

Gini index is a measure of inequality, based on the Lorenz curve. The Lorenz curve is the plot of cumulative proportions of the population ranked by health (from the sickest person to the healthiest) against the cumulative proportion of health. The Gini coefficient is twice the area between the Lorenz curve and the diagonal. It ranges from 0 to 1 (i.e. from complete equality to when all the health is concentrated in the hands of one person) [18]. The calculation of the Gini index for each variable (TB incidence and socio-demographic, economic and structural indicators) was done to distribute the values of these variables in the census tracts. We use the “ineqdec0” function in Stata to obtain the subgroup decomposition for the generalized entropy family [19].

To analyze the determinants of the TB incidence inequality in the neighborhoods, a linear regression analysis was performed, with the Gini of TB incidence in the neighborhoods as an outcome. The explanatory variables analyzed were classified into four dimensions: (i) sociodemographic, (ii) economic, (iii) structural, and (iv) performance of TB surveillance actions.

As explanatory variables of TB inequality, the inequality of the socio-demographic, economic and structural conditions of the neighborhoods of Manaus. The sociodemographic condition was represented by the following indicators: Gini of the proportion of illiterates aged 18 years or older, Gini of the proportion of males, and Gini of the proportion of indigenous population. The indicators that represent the economic condition of the neighborhoods were as follows: mean income per capita and Gini of mean income per capita. The proxies of the structural conditions of the neighborhoods were as follows: Gini of the proportion of households connected to the public water supply network and Gini of the mean number of toilets per inhabitants.

As a proxy for the level of performance of TB surveillance services, the following were used: the proportion of cases with laboratory confirmation (Number of smear-positive patients × 100/number of pulmonary TB cases), the proportion of cases that underwent directly observed treatment - DOT (Number of patients with smear-positive TB on DOT × 100/number of smear-positive TB patients), the proportion of treatment abandonment (Number of TB cases with treatment abandonment information in the 9th month X 100/ Number of TB cases), and the proportion of cure (Patients with at least two negative bacilloscopies, one in the follow-up phase and the other at the end of treatment X 100/ Number of TB cases).

To identify the determinants of TB incidence inequality, a simple linear regression analysis of each explanatory variable and the outcome was performed. Then, the explanatory variables that showed a significance level of 0.2 were analyzed using a multiple linear regression model. Only those variables that showed a significance level of 0.05, selected using the *stepwise backward* approach, were maintained in the final model.

The Stata application (v.13) was used for the analysis and the QGIS application (v.2.18.6) was used for the georeferencing of TB cases and creation of the thematic map.

This study was presented to the Research Ethics Committee of the Adriano Jorge Foundation on June 12, 2017 and was approved (recommendation number: 2.114.304).

## Results

Between 2007 and 2016, 21,030 new TB cases were reported in Manaus, representing a mean annual incidence rate of 79 cases per 100,000 inhabitants. In this period, there was an increase in the disease incidence, from 64.5 cases per 100,000 inhabitants in 2007 to 82 cases per 100,000 inhabitants in 2016.

**Fig. 2:**
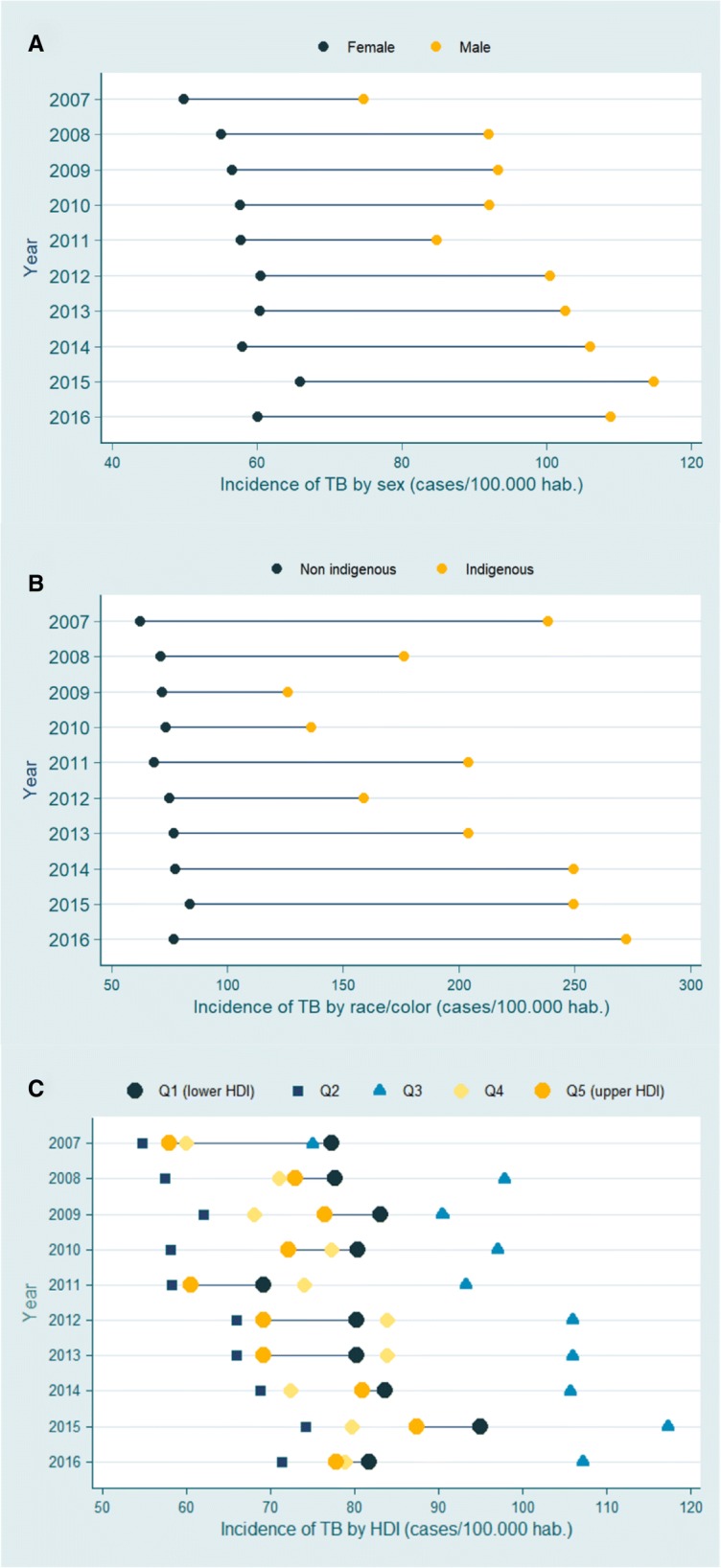
*Equiplot* of tuberculosis incidence in different population groups in Manaus, from 2001 to 2016. **a** gender; (**b**) ethnicity, and (**c**) HDI levels. Note: The points show the mean incidence rate in each population group. The horizontal lines connect the mean incidence of each group. The distance between the points represents the absolute inequality. The greater the line between the two groups, the greater the absolute inequality

The incidence of TB in men was higher than that in women in the entire study period. In addition, there was a growing incidence of TB inequality among men and women, due to the more significant increase of TB incidence in men (Fig. 2a). With regard to ethnic groups, the incidence of TB was higher among indigenous people than in the non-indigenous population. In 2016, the incidence of TB in indigenous people was 274 cases per 100,000 inhabitants, about 3.5 times higher than the incidence of TB among the non-indigenous population. There was also an increase in the TB incidence inequality among indigenous and non-indigenous populations, due to the increase in incidence among indigenous people (Fig. 2b).

When we compared the incidence of TB in neighborhoods with high HDI levels with those with low HDI levels, we observed a higher incidence of the disease in the neighborhoods with lower HDI levels. In absolute terms, the TB incidence in low HDI neighborhoods was 10 cases per 100,000 inhabitants more than in high HDI neighborhoods (Fig. 2c).

**Fig. 3:**
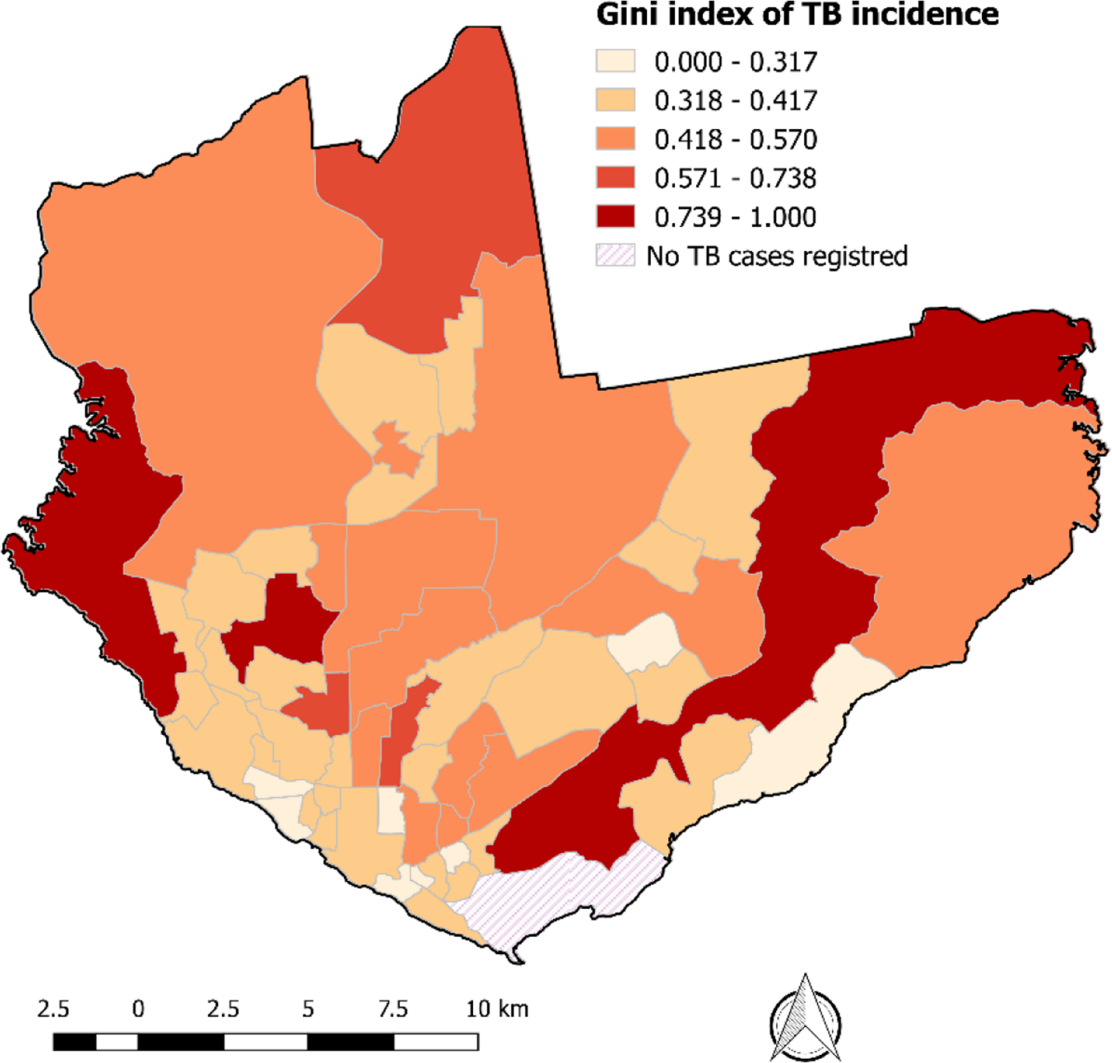
Spatial distribution of the tuberculosis incidence inequality in the neighborhoods of Manaus, from 2007 to 2016

TB incidence inequalities were in the Ponta Negra and Alvorada neighborhoods, both located in the western zone of the municipality; in the Distrito Industrial neighborhood, eastern zone of Manaus; and in the Santa Etelvina neighborhood, in the far north. The lowest levels of inequality were found in the neighborhoods of the southern region of the city, located close to the commercial center of the city (Fig. 3).

The simple regression analysis showed that the TB incidence inequality in the neighborhoods of Manaus is related to the inequality variables studied (Gini of the proportion of male population, Gini of the proportion of indigenous population, Gini of proportion of illiterates, Gini of income, Gini of the proportion of households connected to the water network, and Gini of the mean number of bathrooms per inhabitant), the per capita income, and the proportion of cases with laboratory confirmation. For the latter (proportion of cases with laboratory confirmation), the relation was of inverse type. In the multiple regression analysis, the factors that caused the 43% of variations in TB incidence inequality were as follows: Gini of income, Gini of the proportion of male population, and Gini of the proportion of indigenous population (Table 1).

**Table 1:** The relationship between the TB incidence inequality and the demographic and structural conditions of the Manaus neighborhoods

Factor	Univariate analysis^a^			Multivariable analysis^b^		
	Crude coefficient	*p*-value	95% Conf. interval	Adjusted coefficient	*p*-value	95% Conf. interval
Gini prop. males	1.818	0.001	[0.798–2.839]	1.836	0.001	[0.953–2.718]
Gini prop. Indigenous	0.718	0.011	[0.170–1.266]	0.601	0.011	[0.143–1.060]
Gini illiteracy	0.470	0.001	[0.229–0.711]			
Per capita income	0.001	0.003	[0.001–0.002]			
Gini of income	1.059	0.004	[0.355–1.763]	0.931	0.003	[0.331–1.532]
Gini of water	0.342	0.001	[0.151–0.534]			
Gini of bathroom per inhabitant	1.401	0.001	[0.805–1.998]			
Prop. cases with laboratory confirmation	−0.010	0.008	[−0.018−−0.002]			
Prop. cases that performed DOTS	−0.002	0.217	[−0.007–0.001]			
Prop. abandonment of treatment	−0.003	0.656	[−0.018–0.011]			
Prop. Cure	−0.001	0.929	[−0.009–0.008]			

*Prop* Proportion, *Conf* Confidence, *DOTS* directly observed treatment strategy

^a^ Simple linear regression models; ^b^ Multiple linear negative binomial regression model

## Discussion

From 2007 to 2016, there was an increase in TB incidence in Manaus, accompanied by an increase in the disease incidence inequality among genders, ethnic groups, and socioeconomic level. The disparities in the TB incidence can be explained by the sociodemographic and economic heterogeneity of the population.

TB incidence inequality among genders increased over the study period due to increased incidence in men. These results agree with those observed in several regions of Brazil and the world: men have a higher TB burden than women [1, 20]. This difference in the incidence of the disease between genders may be due to economic, cultural, and social factors [21, 22]. It is known that men have higher consumption of alcoholic beverages and cigarettes [23]. Previous studies have shown that smoking [24] and alcohol consumption [25] are risk factors for active TB. The recognition of the differences in the disease incidence between genders and their tendency to increase indicates the need to plan interventions that consider the differences in the habits and risk factors of each gender.

According to the demographic census conducted in 2010, Manaus had 4406 individuals who declared themselves indigenous, accounting for 0.24% of the population of the municipality. Despite the small proportion, the indigenous population of Manaus had high TB incidence. These results agreed with the findings of research conducted in different ethnic groups, which showed that indigenous populations in the Amazon are at greater risk of acquiring and dying from TB than non-indigenous people [26–29]. In addition to the higher incidence rate, there was a tendency to increase the disease incidence inequality among these racial groups mainly due to the increase in TB incidence rate in the indigenous population. It is common to find among the natives of the Amazon region conditions that favor infection and illness due to TB, such as food insecurity, high prevalence of malnutrition, and intestinal parasitism. In addition, a large part of the non-village indigenous population lives in precarious housing conditions, in poorly ventilated households with low natural light, and has high number of persons per household, which may favor pathogen transmission and the sickness of the infected person [11, 30]. Some researchers suggest that the indigenous population is immunologically susceptible to mycobacteria [27, 31, 32]. Evidence of this is the low frequency of tuberculin skin test reactions to appear by indigenous to the Amazon compared to other populations, even under conditions of high coverage of BCG [27, 32, 33].

The neighborhoods whose population has the lowest HDI levels in Manaus presented, on average, higher rates of TB incidence. These results were consistent with the findings from other investigations. Studies indicated that populations with low socioeconomic level have a higher frequency of contact with people who have active TB; more likely to live in agglomerated and poorly ventilated environments; limited access to healthy food and increased food insecurity; lower levels of knowledge and attitudes related to risk behaviors (such as unsafe sex, smoking, and alcohol use); and difficulties in accessing quality health services [34, 35]. Although there have been fluctuations in incidence rates over time (Fig. 2c), such as lowest rates in 2011 and high rates in 2015, the differences between HDI incidence rates remained consistent.

There were absolute differences in TB incidence among the strata of neighborhoods formed by 1st and 5th inter-quintile intervals of the HDI level in the years studied. This difference persists throughout the study period. These results are consistent with findings from other studies showing the influence of socioeconomic conditions on TB incidence [36, 37]. We observed that neighborhoods belonging to the second interval-interquintile have lower incidence rates than neighborhoods belonging to the other inter-quintile intervals. In addition, neighborhoods belonging to the third-inter- quintile range have higher incidence rates than neighborhoods of the other groups studied. These results were consistent with the findings of another study conducted by our research group [5] that failed to detect a relationship between TB incidence and HDI using a linear regression model, suggesting that variations in the HDI levels does not have a linear relationship with the differences in TB burden among the neighborhoods of Manaus.

We observed that the neighborhoods with high TB incidence inequality, measured by the Gini TB index, are found mainly in the outskirts of the city (north, east and west areas). These are areas of expansion of the city, where it is common to find the installation of new residential condominiums and areas of illegal occupation [38, 39]. The great social contrast in these areas possibly influenced the TB incidence inequality. We found that areas with lower TB inequality have a higher mean incidence of TB than areas with higher TB inequality. In the southern region of the city, we observed a cluster of neighborhoods with lower TB incidence inequality. Despite the lower heterogeneity in TB incidence, the south region had the highest TB incidence rates in the municipality, which may be related to the following sociodemographic conditions: mean number of inhabitants per room, unemployment rate, and proportion of households connected to the sewage system [5].

An association was found between TB incidence inequality and disparities in the structural conditions of the neighborhoods, measured by the Gini of the proportion of households connected to the water supply network and by the Gini of the mean number of toilets by residents. These results indicated that the TB incidence inequality is related to the heterogeneity of the structural conditions of the neighborhoods of Manaus. This heterogeneity of structural conditions made it possible for certain population groups to have a distinct exposure to the pathogen, either due to housing conditions [6] or differences in social relations patterns [7], resulting in different risks of infection and disease progression [40].

With regard to the relationship between TB and socioeconomic inequalities, the findings were similar to those reported by Harling G and Castro MC [4] and by de Castro DB, Sadahiro M, Pinto RC, de Albuquerque BC and Braga JU [5]. Therefore, the disparities in the TB incidence in the city of Manaus could be explained by the sociodemographic and economic heterogeneity of the population. Corroborating the theoretical model that assigns an essential role of the material conditions in the determination of health disparities [41], we observed that there was an association between TB incidence inequality and the mean per capita income of the population of the neighborhoods. Nevertheless, we did not detect such a relationship (TB incidence inequality and mean per capita income) when controlled by the Gini of income index in the multiple regression analysis. This is possibly because the mechanism by which the mean income per capita determines TB inequality was mediated by the income distribution inequality. In Manaus, there was a direct relationship between the mean income per capita and the income distribution inequality (data not shown). Hence, areas with higher mean income per capita had greater income distribution inequality, which in turn was associated to the disparities in TB incidence in the neighborhoods of Manaus.

Another important result of our study was the inverse relationship between the TB incidence inequality and the proportion of TB cases with laboratory confirmation. The proportion of TB cases with laboratory confirmation did not only represent health services coverage, but it was also one of the main quality indicators of TB prevention and control actions [11, 42]. Our findings showed that where there is a better quality of TB control services, there is less disparity in TB incidence.

Our study showed that the greater the income inequality of the population, the greater the TB incidence inequalities among population groups. The Gini of income index is associated with TB inequality independent of other sociodemographic conditions. Ximenes RAdA, Albuquerque MdFPM, Souza WV, Montarroyos UR, Diniz GT, Luna CF and Rodrigues LC [43] argued that the impacts of income inequality on the health of the population may be worse than absolute poverty. Victora CG, Vaughan JP, Barros FC, Silva AC and Tomasi E [44] showed that, in areas with income inequality, population groups with a better socioeconomic level benefit from health resources more than the poor population, accentuating inequalities. To reverse this scenario, the WHO’s Committee on Social Determinants of Health recommended that the analyses of health conditions must be disaggregated by population groups to identify disparities and to support policy-making and the planning of actions focused on the most vulnerable population groups, ensuring the fairness of interventions [45]. It is worth noting that Brazil is the country with the highest inequality of income distribution in the Americas, and the municipalities of the state of Amazonas have the highest Gini of per capita income index in the country [46, 47], making it more urgent to adopt strategies aimed at mitigating health inequalities in the region.

Beyond the material conditions, behavioral and biopsychosocial factors can determine health disparities [41]. Our results appeared to corroborate this hypothesis evidencing the relationship between inequality of TB incidence and heterogeneity of the proportion of male and indigenous individuals in the districts of Manaus. These conditions are associated with TB disparities even after controlling for the effect of income inequality on the population. Psychosocial impacts on the health stemmed from feelings of social exclusion, discrimination, stress, low social support, and other psychological reactions to social experiences [48].

The behavioral effect on health inequalities can be attributed to the differences between social groups or between individuals of the same population in terms of eating habits, smoking habits, or the need to search for health services [49]. These findings reinforced the importance of carrying out studies that may clarify the mechanisms that make these groups more vulnerable to TB and, thus, promote the adoption of effective actions for disease control.

This study had the following limitations: the possibility of underreporting TB cases, either due to problems related to coverage and access to services offered to the population or possible errors of classification and/or diagnosis of TB cases reported in Amazonas. Despite these limitations, the findings remained useful for decision making in public health, since they point out the priority population groups that require interventions to control TB.

## Conclusion

The incidence of TB is heterogeneous in populations that are more unequal in relation to the proportion of men, indigenous people, and groups of lower socioeconomic levels. The disparities in TB incidence can be explained by the population heterogeneity regarding sociodemographic and economic characteristics. Our findings underscore the importance of considering gender, ethnic, and socioeconomic differences in the formulation of public policies and TB control action plans to make them more efficient, equitable, and fair.

## Abbreviations

Conf.: Confidence
DOTS: Directly observed treatment strategy
HDI: Human development index
IBGE: Brazilian Institute of Geography and Statistics
Prop.: Proportion
TB: Tuberculosis
WHO: World Health Organization
